# Characterization of the Cicatrization Process in Diabetic Foot Ulcers Based on the Production of Reactive Oxygen Species

**DOI:** 10.1155/2018/4641364

**Published:** 2018-05-23

**Authors:** Alberto López-Delis, Suélia de S. Rodrigues Fleury Rosa, Paulo Eduardo Narcizo de Souza, Marcella Lemos Brettas Carneiro, Mário Fabrício Fleury Rosa, Yasmin Carneiro Lobo Macedo, Fabiane Hiratsuka Veiga-Souza, Adson Ferreira da Rocha

**Affiliations:** ^1^Medical Biophysics Center, University of Oriente, Santiago de Cuba, Cuba; ^2^Biomedical Engineering Program, University of Brasilia, Brasilia, DF, Brazil; ^3^Laboratory of Electron Paramagnetic Resonance, Institute of Physics, University of Brasilia, Brasilia, DF, Brazil; ^4^Faculty of Planaltina, University of Brasilia, Planaltina, GO, Brazil; ^5^Policy-Making in Collective Health, Faculty of Ceilândia, University of Brasilia, Ceilândia, DF, Brazil; ^6^Laboratory of Biochemistry and Protein Chemistry, Institute of Biological Sciences, University of Brasilia, Brasilia, DF, Brazil; ^7^Faculty of Ceilândia, University of Brasilia, Ceilândia, DF, Brazil

## Abstract

The present study aims at evaluating the correlation between the free radical formation and the healing action of lower limbs' ulcers in a randomized controlled trial with the use of an adhesive derived from natural latex associated with a light-emitting diode (LED) circuit. The sample consists of 15 participants with lower limb lesions divided into three groups: group 1 case (5 participants) received the proposed dressing system adhesive of the natural latex associated with the LED circuit; group 2 control (5 participants) received the dressings at home performed by nurses according to and established by the clinic of wounds (treated with calcium alginate or silver foam); and group 3 (5 participants) also received the dressing in their homes with the use of the dressing adhesive derived from the natural latex associated with the LED circuit. The collected data were analyzed qualitatively and quantitatively by electron paramagnetic resonance for determination of free radical formation. Kruskal-Wallis statistical test was used to evaluate the effect of treatment on the lower limb's ulcer cicatrization process and its correlation with free radical. The results obtained corroborated the hypothesis about the reduction of the quantity of these molecules in the end of treatment related to the healing wound.

## 1. Introduction

The number of people with diabetes has increased from 108 million in 1980 to 422 million in 2014. The global prevalence of diabetes in adults (over 18 years) has increased from 4.7 percent in 1980 to 8.5 percent in 2014. The incidence of diabetes has increased more rapidly in low- and middle-income countries [1]. This pathology is of silent evolution and develops complications such as retinopathy, nephropathy, neuropathy, and cardiovascular and vascular diseases [1]. Among the difficulties, it is concerned with vascular and neuropathic complications that contribute to the formation of lower limb wounds [2].

In this regard, these vascular and neuropathic complications often evolve into lower limb wounds that are a severe problem in public health. Among the comorbidities, the etiology of the diabetic foot is a multifaceted pathophysiological condition characterized by ulcers, infection, or destruction of the deep tissues of the feet in people with diabetes mellitus (DM). The above occurs because of neuropathy, peripheral vascular disease, or deformities in the lower limbs [3]. The diabetic foot is one of the most devastating complications of DM since it may lead to ulcerations that potentially end up in minor to major amputations. The healing process of wounds has different stages, starting from the bedsores to the restoration of the damaged tissue. These stages are hemostasis, inflammation, proliferation, epithelialization, and scar maturation (repair) [4, 5].

In this respect, it is fundamental to choose the correct methods to treat the wound better and to assess healing effectiveness. One example is the use of new coatings, among them the latex derived from *Hevea brasiliensis* in animals [6–8] and humans [9–12]. In a study, Quege [13] analyzed the wound healing efficacy of an essential fatty acid dressing in 11 participants and of a treatment based on a natural latex (Biocure) coating in 8 participants. We noticed that, in both procedures, there was a decrease in the size of the wound with slightly better improvement in the individuals treated with Biocure. Brandão et al. [14] reported the development of a new microperforated vascular graft model made of tissue and covered with a natural latex compound that showed good structural qualities as a vascular substitute, which stimulated endothelial growth and provided adequate tissue integration in dogs.

The LED circuit, associated with latex, was also investigated by Reis [15, 16], who compared the treatment of four participants with silver foam dressing with a treatment of six other participants with latex in association with the LED light for 30 minutes. The clinical findings were analyzed qualitatively and quantitatively, demonstrating that the results obtained by the experimental group were better than those obtained by the control group; the results suggested that the system proposed is a promising treatment option for diabetic foot ulcers, due to its healing properties. The use of LED circuit was also tested with other healing products introduced by Caetano [11], who evaluated the treatment of venous ulcer with silver sulfadiazine, by using the dressing in association with the LED circuit in 7 participants of the research, on a daily basis. Another group (14 participants) used silver sulfadiazine twice a week, also associated with the LED light. Both groups obtained satisfying results; however, the participants of the study that received light emitted at 891 nm showed a better evolution in the healing of the ulcers.

The recent growth in the awareness of free radicals and reactive oxygen species (ROS) in biological studies is bringing about a medical revolution that promises a new age of health and disease management [15]. Most of the potentially harmful effects of oxygen are due to the formation and activity of several chemical compounds, known as ROS, which tend to donate electrons to other substances. Free radicals and antioxidants have become commonly used terms in modern discussions of disease mechanisms [15]. Free radicals and other ROS are derived either from standard essential metabolic processes in the human body or from external sources such as exposure to X-rays, ozone, cigarette smoking, air pollutants, and industrial chemicals [15]. Free radical formation occurs continuously in the cells as a consequence of both enzymatic and nonenzymatic reactions.

Hence, the use of new coatings such as latex biomembrane and other dressings associated with LED, has been shown to be an option for wound healing in the lower limbs. The healing method used in this study is based on the device named RAPHA, and it consists in light-emitting equipment that has a cluster of 30 high-brightness LEDs in red color, aiming at promoting wound healing by stimulating the patient's tissues, regardless of the underlying cause of the ulcer. The treatment protocol with the equipment has a 35-minute duration. The LED phototherapy method in association with the latex biomaterial is noninvasive and nondestructive. The exposition of the light generated by LEDs accelerates cell growth. A set of LEDs that emits light in the visible spectrum increases the energy of the cells, which speeds up the healing process of patients. LED therapy is based on the fact that light can alter cellular metabolism as a result of its absorption by mitochondria.

This survey aims at examining the correlation between the free radical formation and the healing action of lower limb ulcers in a randomized controlled trial by using a natural latex-derived adhesive associated to a LED-based circuit. The main goal of this study is to demonstrate the advantage of the use of LED and latex biomembrane in the healing of ulcers. Additionally, the results may pave the way for a change in the current treatment, by decreasing treatment and healing duration in ulcers.

## 2. Materials and Methods

### 2.1. RAPHA Equipment

It is a mobile tissue neoformation system based on the phototherapy principles to assist in the healing of wounds. Its LED light-emitting circuit consists of two modules, a control module and a LED module. The tissue neoformation induction system aims at treating patients who suffer from ulcers caused by various diseases and do not respond well to conventional treatments. This system may be seen as a new form of phototherapy, with reduced costs, due to the use of high-brightness LEDs instead of a laser.

The latex sheet has been made with centrifuged 60% latex biomaterial [15, 16]. In the manufacturing process, the latex was placed in a glass or an acrylic Petri dish, previously cleaned and dried, where the latex biomaterial was spread to form a thin layer, covering the dish surface. The Petri dish was not left horizontally in the oven—it was entirely rested in a vertical position so that any latex excess would be drained [15, 16]. This procedure led to a more transparent sheet. We repeated the process six times, and the resulting final sheet thickness was 0.5 mm.

The latex sheets were cut in rectangular shapes and then sterilized with ethylene oxide. We made layers with different sizes to match different ulcer sizes [15]. Purposely, we made the sheets slightly larger than the extent of the wound, to ensure the total covering of the ulcerated area. The latex sheets were tested at the Optical Spectroscopy Laboratory of the Institute of Physics at the University of Brasilia, to verify if it does not cause excessive attenuation or significant changes in the spectral characteristics of the light irradiated by the LED matrix [15]. The results showed that the sheet causes a reduction of approximately half in the intensity of the light but did not affect its spectrum [15, 16].

### 2.2. Subjects

The survey was conducted on the premises of the Ceilândia Regional Hospital's ambulatory clinic (Brasilia, Brazil) and at the residence of the participants of the experimental groups. The subjects of the survey previously provide the written consent, give the permission for publication, and share their photographs for scientific and educational purposes. The study has been approved by the Human Research Ethics Committee of the Health Science Teaching and Research Foundation (process number 52305715.6.0000.5553/2018), Brasilia, Brazil.

We chose as a reference site the ambulatory clinic in Ceilândia, at the clinic for treating chronic wounds. At first, we selected the participants of the survey at the ambulatory clinic; then, they were visited in their houses to carry out the other phases of the research (according to the three specific groups). All the participants received the proper information and signed the free and informed consent form.

The sample group consisted of 15 individuals, divided into three groups of 5 participants of the study, regardless of their gender. The sample group presents individuals with lower limb ischemia and neuropathic ulcers. The fifteen participants with lower limb lesions were divided into three groups (the data are presented in the mean ± standard deviation format):

- Group 1 (GI) case: 5 participants treated by qualified nurses with the dressings at the ambulatory clinic using the application of latex in association with the LED circuit (age: 61.8 ± 11 yrs; height: 1.65 ± 0.09 m; weight: 90.7 ± 39.5 kg)
- Group 2 (GII) control: 5 participants treated at home by nurses, following the standard wound care recommendations (with silver alginate dressing) (age: 62.6 ± 8.54 yrs; height: 1.54 ± 0.05 m; weight: 70.86 ± 14.4 kg)
- Group 3 (GIII): 5 participants self-treated at home with the dressing in association with the natural latex adhesive and the LED circuit (age 55.4 ± 10.3 yrs; height: 1.67 ± 0.08 m; weight: 81.4 ± 11.6 kg).

To participate in the survey, the participant should meet the following criteria:

(1) Received the treatment on the premises of the Ceilândia Regional Hospital
(2) Participated in the treatment adherence group and sign the informed consent form (TCLE)
(3) Participated in a previous survey on the healing of lower limb ulcers
(4) Had a lower limb ulcer caused by neuropathic or vascular diseases with or without clinical signs of infection

The participant of the research should not

(1) refuse to be part of the research group;
(2) be pregnant, underage, or over 75 years old;
(3) consume alcoholic drinks or illicit drugs;
(4) have evidence of osteomyelitis or gangrene on the affected extremity;
(5) topically apply any medication at the wound site not adopted in this protocol after the start of the trial;
(6) miss the treatment program (for three consecutive times).

### 2.3. Devices Used

**Figure 1:**
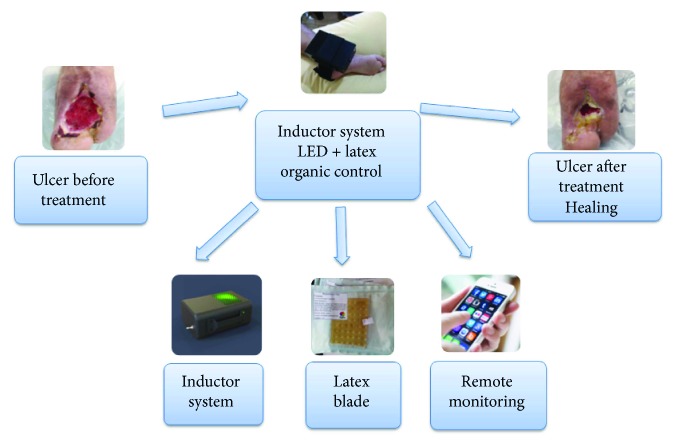
Flowchart for the application of the randomized clinical trial of research participants with wounds: natural latex and light-emitting circuit of LEDs.

The method proposed for this prospective research is the simultaneous action of the natural latex adhesive and the LED circuit. Both agents have characteristics and properties capable of inducing tissue regeneration and neoformation.  Figure 1 presents the flowchart for the application of the randomized clinical trial research with wounds: natural latex and LED circuit.

The latex extracted from the Brazilian rubber tree is a polydisperse system, in which negatively charged particles of various types are suspended in serum due to its neovascularization- and tissue regeneration-inducing properties. Natural latex is healing, for it is a natural defense of the plant. It is of natural origin and presents a low cost, without risk of transmitting pathogens and with great clinical-social applicability.

The process of making the adhesives is done by mixing the bicentrifuged latex with doubly distilled water, placing the mixture in a previously sterilized mold, selected with the approximate dimensions of the wound. This mixture is then accommodated in the mold without leaving extra material or creating air bubbles. After the abovementioned processes, the material undergoes a vulcanization process inside a greenhouse. The adhesive is then removed from the mold and undergoes a sterilization and packaging process before being used by a participant of research.

The LED circuit consists of two modules, a control module and a LED module. The control module has a timer to indicate the duration of light emission that beeps at the end of the procedure. It is directly connected to the module of LEDs that has 30 monochromatic LED lights. The system assists the wound healing process.

### 2.4. Sample Collection Protocol

All sample solutions were prepared immediately before the experiments in Krebs HEPES buffer (KHB), pH 7.4, dissolved in a bidistilled and deionized water. Ten mM stock solution of CMH was freshly made and dissolved in KHB containing 25 *μ*M deferoxamine methanesulfonate salt (DF) and 5 *μ*M sodium diethyldithiocarbamate trihydrate (DETC). The solutions were kept cooled throughout all the measurements.

**Figure 2:**
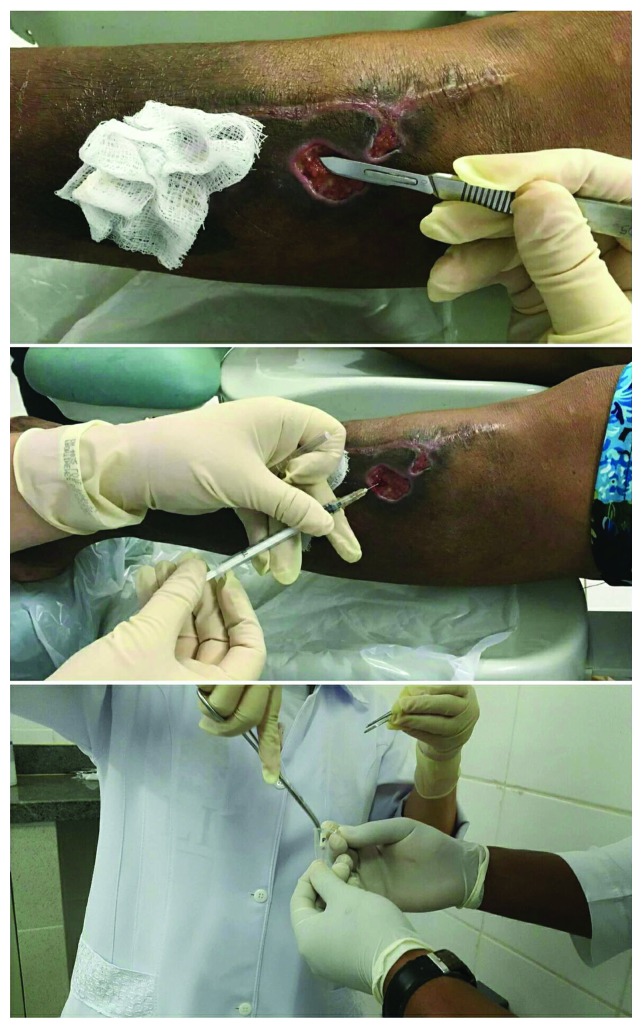
Collection of wound tissues on tissue samples before treatment and collection of blood in the wound bed (50 *μ*L).

Two samples of the wound and venous blood, respectively, and one collection of injured tissues were taken from the subjects to evaluate the healing process (Figure 2). For venous blood collection (1.5 mL), we used a syringe or a heparinized tube. The site of preference for venipunctures is the antecubital fossa, in the anterior area of the forearm and below the elbow, where there are many veins, relatively close to the surface of the skin. First, the patient's arm was set up, by tilting it down from shoulder height. The blood was collected slowly, with a maximum of 40 mmHg of compression in the arm. After removal, the material was frozen in liquid nitrogen.

For the blood collection (2 to 3 drops), we collected a sample from the edges from small holes, where there was forming tissue. We used a syringe or heparinized tube with a special needle (as of insulin, smaller caliber) and deposited the blood in heparinized microtubes. After removal, the material was frozen in liquid nitrogen.

Finally, the collection of wound tissues was accomplished (tissue samples before treatment and granulation tissue formed during healing). The material was collected by scraping (close to the edges) with a sterile wooden spatula. Then, the wooden spatula was hermetically sealed in a tube and frozen in liquid nitrogen.

We established three stages for sample collection: on the first day before the beginning of the treatment, on the second day after two weeks of treatment, and at the end of treatment, around 30 days after the start of the treatment (or sooner, if the wound is healed).

### 2.5. Detection of ROS in Human Blood

The wound and venous blood were thawed, and 50 *μ*L samples were treated with a solution containing 400 *μ*M 1-hydroxy-3-methoxycarbonyl-2,2,5,5-tetramethylpyrrolidine (CMH) and heparin sodium (100 IU/mL) in 1 : 1 proportion. The tube was incubated under gentle shaking at 37°C for 30 min. After that, 50 *μ*L of the obtained solution was placed between two ice blocks (200 *μ*L each) in a one-mL decapped syringe and snap frozen in liquid nitrogen. All samples were stored at −80°C until the electron paramagnetic resonance measurements were performed. The principle of the method is based upon the ROS interaction with CMH to form a stable radical 3-methoxycarbonyl-2,2,5,5-tetramethylpyrrolidine-1-oxyl (CM^·^), detectable by EPR, which is a signature of ROS generation.

### 2.6. Electron Paramagnetic Resonance (EPR)

**Figure 3:**
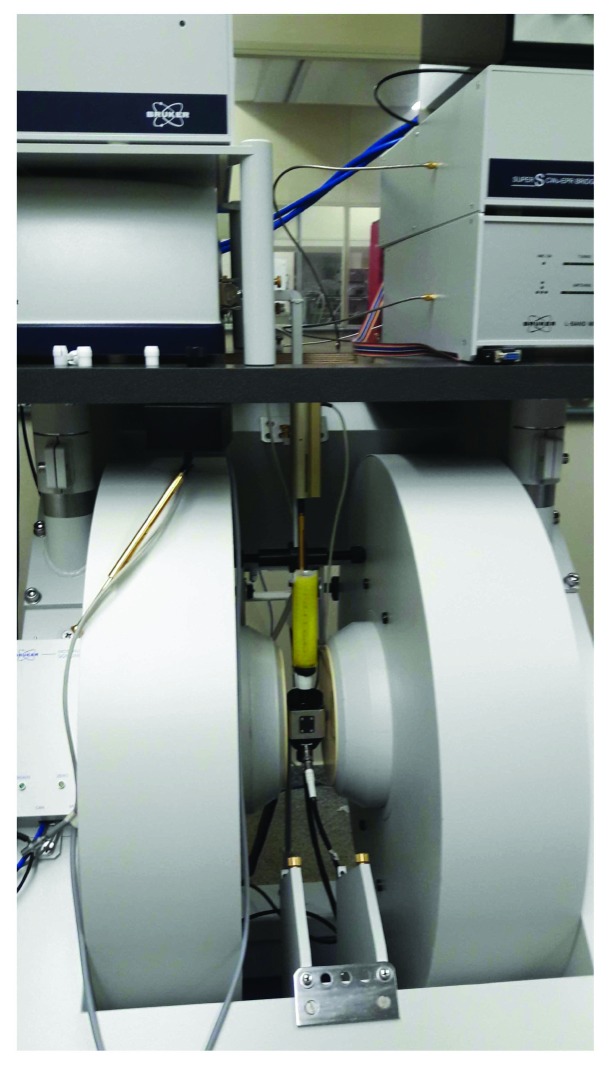
Electron paramagnetic resonance spectrometer EMXplus (Bruker, Germany).

EPR measurements were performed in an EPR spectrometer EMX plus (Bruker, Germany), by using X-band (9 GHz) and a high-resolution cavity (ER 4119HS, Bruker, Germany), as illustrated in Figure 3. For ROS detection, the samples were transferred to a liquid nitrogen Dewar (Noxygen, Germany), and the spectra were recorded at 77 K. The instrumental settings were two-mW microwave power, 5G modulation amplitude, 100 kHz of modulation frequency, and 200G sweep width.

We used peak height for detecting the signal. The peak height is the arbitrary distance between lowest and highest point in the first derivative spectrum. The calibration curve was made by using the nitroxide radical CP^·^ diluted in KHB at a dose range of 0, 5, 10, 50, and 100 *μ*M. In this concentration range, a linear calibration curve was obtained, and all the recorded data were within this calibration range.

### 2.7. Chemicals

The spin probe 1-hydroxy-3-methoxycarbonyl-2.2.5.5-tetramethylpyrrolidine (CMH); the metal chelators deferoxamine (DF) and diethyldithiocarbamate (DETC); Krebs–HEPES buffer (KHB); and the stable radical CP^·^ (3-carboxy-2,2,5,5-tetramethyl-1-pyrrolidinyloxy) were obtained from Noxygen Science Transfer & Diagnostics.

### 2.8. Statistical Analysis

Statistical analysis was performed by Matlab (Mathworks Inc., Natick, MA). The micromolar concentration metric related to CM^·^ was used to evaluate the real effect of the treatment. The methodology applied for this verification was the Kruskal-Wallis test. This test is a nonparametric version of the classical one-way ANOVA and an extension of the Wilcoxon rank-sum test to more than two groups [17]. It compares the medians of the groups of data to determine if the samples come from the same population (or, equivalently, from different populations with the same distribution). The *F*-statistic used in classical one-way ANOVA is replaced by a chi-square statistic. The *p* value measures the significance of the chi-square statistic, for this case with a significance of 1% [17].

## 3. Results

### 3.1. EPR Measurements

**Figure 4:**
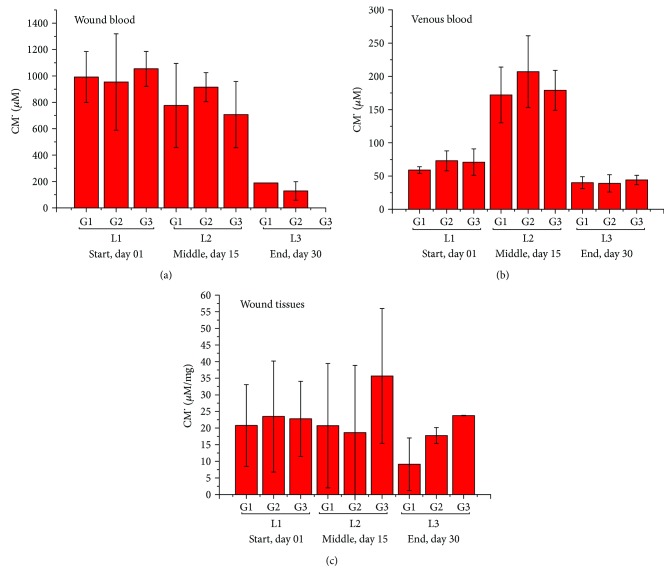
CM^·^ quantification in the wound (a), venous blood (b), and wound tissues (c).

Figure 4 presents the EPR result of ROS quantification from CM^·^ in venous (a) and wound (b) blood from the lesions of diabetic patients and wound tissues (c).

In general, the results showed that all experimental groups (G1, G2, and G3) presented similar amounts of ROS in the wound blood considering the same period of analysis (days 01, 15, or 30). Nevertheless, when comparing each experimental stage (days 01, 15, and 30), the results showed a pronounced reduction of CM^·^ between days 01 and 30, which correspond to the beginning and the end of the treatment (Figure 4(a)).

Regarding CM^·^ data in the venous blood, it was also noticed that G1, G2, and G3 groups presented similar amounts of ROS when comparing each isolated analysis period. However, it was also observed that the ROS concentration was different between days 1 and 15 and between days 15 and 30, considering the same experimental group. Thus, we verified the increase of venous blood ROS formation in the middle of the treatment (day 15) in comparison to the beginning (day 01) and the end (day 30) of the treatment period (Figure 4(b)).

According to the analysis of CM^·^ content in wound tissues, there was a statistical difference among the three experimental groups at all times evaluated. Therefore, there was a variation in the generation of ROS in the wound tissues between the beginning and end of treatment (Figure 4(c)).

### 3.2. Statistical Performance

**Figure 5:**
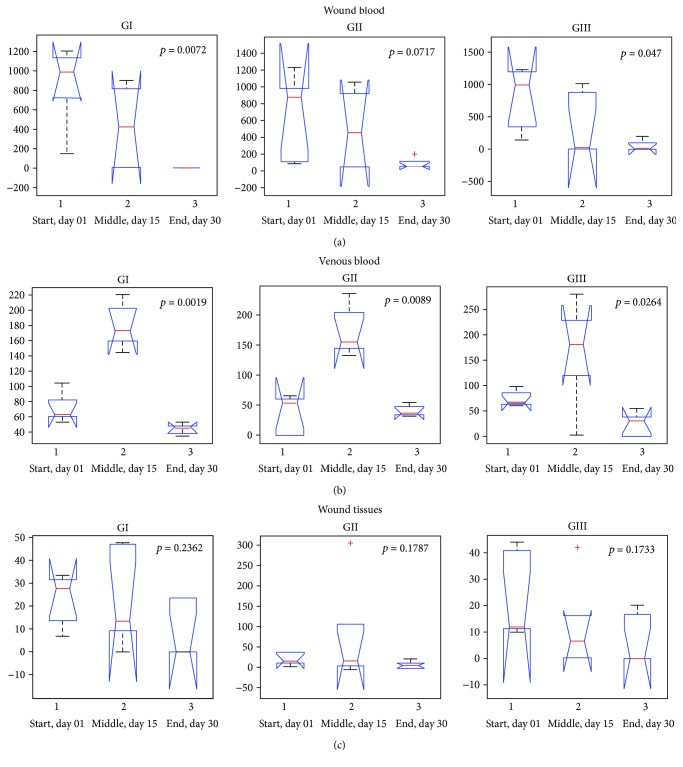
The statistical dispersion obtained along the sample collection stages in the three sample groups with statistical signification in the wound (a), venous blood (b), and wound tissues (c).

Figures 5(a)–5(c) present the statistical dispersion obtained along the sample collection stages in the three sample groups with statistical representation in the wound and venous blood as well as in the wound tissues.

## 4. Discussion

We identified high concentration of CM^·^ in the wound blood at the start and at the middle of the treatment (Figure 4(a)), which is evidence closely related to the inflammation process. The significant reduction in ROS formation at the end of the treatment (Figure 4(b)) is associated with the wound healing process since the decreasing of ROS levels is related to the reduction of the inflammatory process (see statistical dispersion in Figure 5(a)). ROS act as inflammation signaling molecules, and they are involved in the host-defense response [18–20]. Neutrophils, macrophages, and immune cells mediate immune reactions employed in the “oxidative burst,” which is characterized by the rapid production of large amounts of intracellular ROS. Moreover, the exposure of these cells to proinflammatory cytokines, during the inflammatory process, induces a significant increase of the ROS levels into the immune cells, boosting the ROS production [18, 19]. During the first 15 days of the treatment, a high generation of ROS was identified in the wound blood (Figure 4(a)), corresponding to the inflammatory process which precedes the wound healing.

By the end of treatment, at about the 30th day, we noticed a significant reduction of ROS generation in the wound blood (Figure 4(b)), which is related not only to the decline of the inflammation but also to the induction of the apoptosis in wound cell remnants.

Apoptosis, or programmed cell death, is closely related to the control of the swelling, and it has an essential role in the advancing epithelial edge toward the center of the wound, evidencing healing progress. The wound healing involves a series of rapid increases in specific cell populations that prepare the wound for repair, deposit new matrices, and, finally, mature the wound. For this reason, these types of cells must be eliminated from the injury before the progression to the next phase of the healing, triggered through apoptosis, which allows the eliminations of entire populations without an inflammatory response or tissue damage [18–20].

It is widely reported that ROS behave as signaling molecules for inflammation in the endothelial dysfunction. Under the inflammatory conditions, oxidative stress, produced by immune cells, leads to the opening of interendothelial junctions and promotes the migration of inflammatory/immune cells across the endothelial barrier. The immune cell emigration elicits the acute inflammatory response essential to repair injury tissue [18, 19]. Thus, the increase of ROS formation, observed in the patient venous blood in the middle of the treatment period (day 15, Figure 4(b)) could be related to the endothelial dysfunction and to mobilization of immune cells to the wound area. The mechanism described results in higher ROS formation in the venous blood in the 15th day (see statistical dispersion from Figure 5(b)). Similarly, there was a variation in the amount of ROS in the wound tissues between the beginning and end of treatment (Figure 4(c), see statistical dispersion in Figure 5(c)). In particular, in groups G1 and G3 (patients that are receiving latex and LED treatment), a reduction in wound ROS concentration at the end of the treatment (30th day, Figure 4) was observed, which corresponds to a decrease of the inflammation. It is related to the roles of ROS antioxidants in skin wound healing, their possible involvement in chronic wounds, and the potential value of ROS-induced biomarkers in wound healing prognosis [21].

Together, these results suggest the existence of modulation of environmental oxidative stress in chronic wounds and their contribution to healing in these scars. It is known that the ROS generation is critical for the inflammatory modulation response and plays a significant role in the impact on several functions in the cells. There are multiple sources of ROS, both inside and outside the cell, and diverse ROS species that are generated in different zones within a cell, at different times and at different concentrations. All these factors significantly influence the effectiveness of ROS and present distinct results [19]. Thus, the understanding of the effect of ROS generation in the wound blood, venous blood, and wound tissues, conducted in this survey, was essential to elucidate its biological effects as a target molecule in the healing wound.

Moreover, the participation of the nurses was fundamental to the evaluation of the wound. For this process, elements such as the degree of healing and the sample collection site can affect the sample data (ROS equal to zero) and the nurse evaluation in avoiding injuring the newly formed area. In this analysis, we noticed that the reduction of the pH of bedsore stimulated the production of cytokines and the reproduction of these cells, thereby reducing the growth of pathogenic microorganisms. We also concluded that the system acted as debridement and that it had an anti-inflammatory action, as evidenced in the images obtained in the analysis of the contraction and the junction of borders of the healing wounds.

We concluded that this system could be implemented in various healing stages, with different concentrations, according to the type of the wound tissue (this is the next step of the study). In this context, it is necessary to evaluate the best options for data collection, considering its effectiveness.

We must also take into consideration that, in clinical practice, the evidence presented in this study contributes to the decision making for the treatment approach, suggesting that it can be done by modifying the light intensity, the duration of exposure, and the thickness of the latex adhesive according to the stage of the healing process. The granulation tissue observed for the applied light intensity and the characteristics of the lamina and when there is a presence of necrosis of innovative liquefaction approach of intensity and time of use of the lamina must be incorporated and finally should be performed in coagulation necrosis and escharotomy.

There were no side effects observed in this survey, although the participants reported burning and pain during and after the treatment. Considering that it was a subjective perception, there was no need to specify the concentration and intensity of climbing point for future studies. The system (latex and phototherapy) yielded better good debridement and acceleration of the healing process with the formation of granulated tissue, when compared to the current standard procedures.

We also point out the necessity of training the nursing team on the manipulation of the product, to collect samples and especially to manage the patient's ability to obtain samples of wounds already healed. The use of latex demonstrated a maceration of the wound edges and a granulation tissue susceptible to fragmentation due to its softened feature. The system presented in this study can be applied in different stages of the healing process in diverse etiologies, but it still requires further investigation.

We concluded that high ROS formation in the wound blood, in the beginning and in the middle of the treatment, correspond to the inflammation process and that the reduction of the quantity of these molecules by the end of the treatment is related to the healing process of the wound.
